# Research on a Denial of Service (DoS) Detection System Based on Global Interdependent Behaviors in a Sensor Network Environment

**DOI:** 10.3390/s101110376

**Published:** 2010-11-17

**Authors:** Jae-gu Song, Sungmo Jung, Jong Hyun Kim, Dong Il Seo, Seoksoo Kim

**Affiliations:** 1 Department of Multimedia, Hannam University, Daejeon, Korea; E-Mails: bhas9@paran.com (J.-G.S.); sungmoj@gmail.com (S.J.); 2 School of Computing & Information System, Tasmania University, Hobart, Australia; 3 Electronics and Telecommunications Research Institute, Daejeon, Korea; E-Mails: jhk@etri.re.kr (J.H.K.); bluesea@etri.re.kr (D.I.S.)

**Keywords:** sensor network, dos attack, interdependent behaviors, security

## Abstract

This research suggests a Denial of Service (DoS) detection method based on the collection of interdependent behavior data in a sensor network environment. In order to collect the interdependent behavior data, we use a base station to analyze traffic and behaviors among nodes and introduce methods of detecting changes in the environment with precursor symptoms. The study presents a DoS Detection System based on Global Interdependent Behaviors and shows the result of detecting a sensor carrying out DoS attacks through the test-bed.

## 1. Introduction

The number of security breaches is on a sharp increase and so is are the damage and losses. Although the actual amount of damage from malicious codes has not been fully revealed, it is enormous, and such damage occurs from common services such as in cases of game hacking, messenger phishing, voice phishing, and so on [1]. Moreover, previous methods of cyber attacks have begun to use wireless sensor networks, calling for varied research on protection methods. Particularly, the previous methods of attacks used in wired networks can be applied in the same manner with sensor networks. For instance, it is difficult to detect and respond to such an attack due to the mobility of wireless network clients and independent operation in an open environment [2].

Sensor networks have already been used along with a smartphone, offering various applications in fields as diverse as the medical, military, environmental and entertainment services in a multitude of areas and, thus, DoS attacks using the environment are likely to cause tremendous damage.

Therefore, we need to analyze cases of DoS attacks showing various patterns and develop a detection method to respond to attacks using the sensor networks. Currently, most research on sensor network security focuses on key distribution and management, authentication, network structure, routing, and so on, but there is lack of research on precursor symptom detection.

In this research, therefore, we’ve carried out research on precursor symptom detection to cope with DoS attacks in sensor networks. For that reason, we have studied varying vulnerability in an existing sensor network and, based on the results, presented an interdependent-based DoS detection system that can predict vulnerability.

In order to verify interdependent behaviors, this research is based on a structure which includes a base station and aggregator. Traffic changes and packet data were also analyzed by means of node data management.

## 2. Basic Research

A sensor network randomly detects an object in a limited area and sends this information to a base station. This means a user is exposed to an environment in which the data are highly likely to become redundant or get lost due to the numerous nodes involved.

Due to its nature, a sensor network is more vulnerable to cyber attacks than a wired network. In particular, the open environment makes it more difficult to prevent disclosure of information. As a result, one may easily modify data or allow a malicious node to transmit data, destroying the integrity of the entire data or causing excessive load on the network. Particularly, in WSNs, nodes are not controlled once they have been arranged so that a malicious user may destroy, capture, or compromise the nodes [3].

Common attacks using the wireless network include the capture/compromise of nodes, interception, DoS, and router attacks such as HELLO Flood, Sinkhole, Wormhole, and so on. Each type of router attack is defined as below:

▪ Sybil: It causes a node to recognize a single node as a number of identifiers, fatal to geographic routing [4].
▪ Hello Flood: A remote attacker sends a HELLO packet with a strong signal so that a packet can be sent to the attacker [5].
▪ Wormhole: A node connection, which does not exist, is recognized, used for interception or selective forwarding [6].
▪ Sinkholes: It is used along with selective forwarding for interception. The routing data are modified and all the data are induced to pass through the attacker’s sinkhole [7].

A cyber attack in the sensor network may develop into the use of one more than one method of attack. That is, one may use a wireless jamming attack for DoS or a battery exhaustion attack.

Attack methods in wireless sensor networks are described as follows:

▪ Sniffing (Interception) [8]: A sensing data message or signal message in a wireless channel could be intercepted or exploited to be analyzed for other attacks.
▪ Battery exhaustion attack [9]: An attacker causes a battery to be used up in a short time so that sensor nodes can not be used anymore. To that end, he may keep requesting data transmission or network connections. A PDoS (Path-based DoS) attack, recently analyzed, shows that a huge number of bug packets are flooded toward the base station in order to induce fast exhaustion of a battery of nodes, reducing the life of the nodes [10].
▪ Wireless jamming (signal interference) [11]: An attack of jamming a frequency band or paralyzing a communication channel by continuously sending a signal.
▪ Physical tampering and side channel attacks [12]: Physical tampering refers to an attack of destroying or dismantling device hardware while a side channel attack means a method of analyzing electric signals from a sensor node or analyzing other signals such as consumption of power. This attack is fatal, for it uses an extracted security key, affecting the entire sensor network.
▪ Routing attack [13]: False routing data could be provided by a sensor network based on a broadcast network and then routing protocols fabricated. A routing message received could be spoofed, modified, or re-sent, disturbing routing and thus delaying generation or transmission of a routing loop.
▪ Denial of Service (DoS) in the sensor network [14]: Sensing data services of the sensor network are real-time context-aware services and vulnerable to DoS when an attacker disturbs routing or a message attack delays processing and transmission time, making meaningless real-time services. Common patterns of attacks include launching attacks on a sensor node or BS by means of various methods, blocking transmission of sensing data or causing an error in control signals, which makes services impossible to be offered.
▪ IP Spoofing [15]: An IP-based sensor node or gateway node is an IP-based network so that an attacker may disguise himself as an authenticated user of sensor services in order to attack a sensor node or network.

Attacks exploiting vulnerability in protocols or OS include examples such as a Trojan virus, worm, malicious code, virus, and so on [16].

In an IP-based sensor network or sensor node, an attacker may use a communication channel for an IP network or a control channel in a reverse direction so as to distribute vulnerability of OS, a worm, a virus, a malicious code, and so on. Using some vulnerability in the OS or protocols, such a virus can paralyze sensor nodes, intercept security information of the sensor network, or capture sensor nodes in order to develop a bot and, eventually, attack the entire network.

## 3. Interdependent Behaviors-Based DoS Detection Method

### 3.1. Tracking Behaviors between Sensor Nodes

The most effective method of identifying a malicious node in the communication between nodes of the sensor network is to collect data of nodes communicating with the base station. Before the base station accepts a request from a node, the behavior of a node is analyzed and a malicious node is not included in the communication, alleviating DoS attacks. To do so, behaviors between sensor nodes shall be tracked. First, it is supposed that all the nodes regularly send data to the base station [17].

When a sensor node generates and sends data, looking for a routing path, it specifies the nodes that have passed by while a packet header arrives at a target node. Also, a malicious node can be tracked by counting a hop node that is generated continuously along the routing path. In this research, we send data, collected by constructing an *ad-hoc*/multi-hop network between application nodes, to a base node and analyze them.

### 3.2. Traffic Analysis

We have categorized traffic flowing into a wireless network into several patterns by analyzing the data traffic transmitted by nodes. In this research, data traffic between nodes was analyzed by applying the DEWP (Detecting Early Worm Propagation through Packet Matching) [18] research, which has been presented for DoS detection. This method detects a sudden increase in traffic created by a specific node or an abnormal amount of traffic compared to previous traffic generation. For the reason, we have compared the amount of packets sent to a specific node during defined time and the amount of packets sent out of that node. That is, we used the EWMA (Exponential Weighted Moving Average) algorithm in order to compare the amount of packets sent to a specific node during t-time and the amount of packets sent out of that node.

The change in traffic analyzed by the EWMA algorithm is as follows:

(1) [K′=α×K′+(1−α)×K]

where K refers to the number of addresses (sources) of the traffic flowing into a specific node while K′ means the average.

If K > K′ × (1 + σ) is satisfied, it means that abnormal traffic has occurred, and the traffic shall be blocked; σ refers to the standard deviation α.

As to global interdependent behaviors, nodes are extracted by studying patterns and mechanism of malicious behaviors based on the results of tracking behaviors between nodes and traffic analysis as mentioned above. This method has been known to be most effective, for its detection is based on the mechanism of a malicious code. A series of behavior rules are compared with interdependent system requests and the result is used for allowing a service or a system, which is applied to blocking, interconnection, engine control, and so on.

This method applies rules and can detect new patterns based on the previous mechanism but it requires constant monitoring for infection between nodes due to host-based detection.

**Figure 1.**
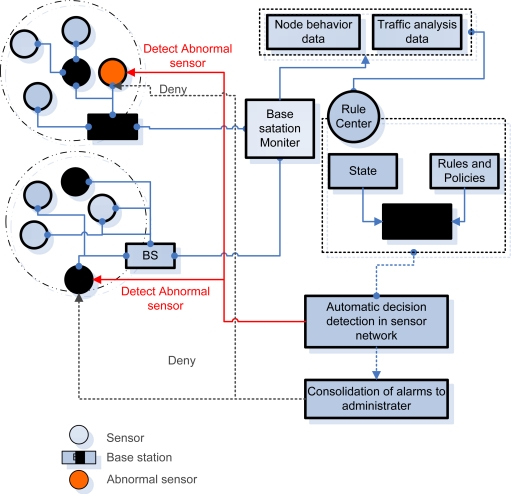
DoS detection system based on global interdependent behaviors.

Figure 1 shows the structure of the DoS detection system based on global interdependent behaviors. The *ad-hoc* sensor network monitors data and traffic information at each BS while BSM, which monitors BS, analyzes all the data related with behaviors and traffic in order to send them to the rule center. Based on the data on the conditions of a node, rules and policies are applied so as to detect an abnormal sensor node and report it to a manager as a precursor symptom. Finally, the node manager confirms the abnormal sensor and removes it from the *ad-hoc* network.

In this research, five requirements presented by the requirements of an effective detection system [19] are considered.

▪ Multiple detection mechanisms
▪ Attack Coverage
▪ Granularity of Attack detection
▪ Consolidation of alarms
▪ Response Action

## 4. Scenario and Implementation of Test-bed

**Figure 2.**
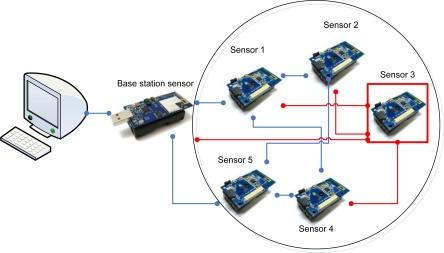
Test-bed environment.

In this study, a base station and five nodes are used in order to monitor data transmission and traffic conditions in a normal environment. The data and traffic are analyzed when a node attempts to keep sending a specific message to other nodes. Figure 2 shows the structure of the test-bed.

Sensor 3 requests sensing data of each sensor per 0.1 second and sends the data to BS. Here, the address of the sensor requested is randomly selected among 1, 2, and 4. Also, it requests abnormal data (a command that can not be responded) from nodes, causing traffic and load on the entire sensor nodes.

The total number of nodes is five and BS allows a PC to monitor. Node 1 and Node 2 generate traffic per 0.5 second by means of data transmission while Node 4 and 5 generate traffic only there is a change in sensing values, Node 3 requests sensing values from Node 1, 2, and 4 while constantly sending data to BS.

### 4.1. Traffic Analysis Data

This traffic analysis data makes clear the difference between ordinary sensor node and abnormal sensor node when abnormal sensor request malicious messages.

**Figure 3.**
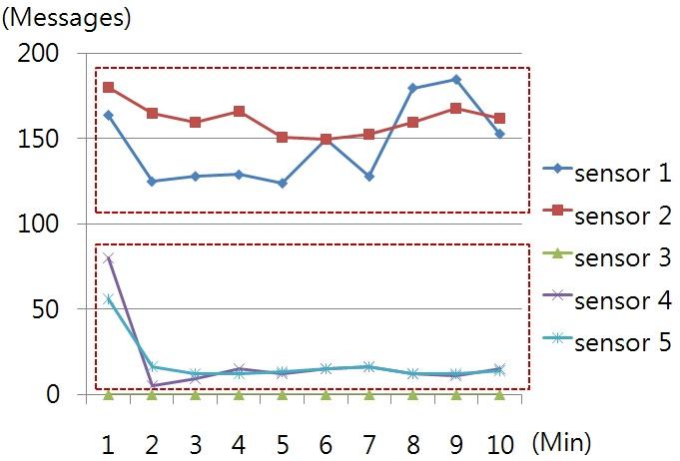
Normal message transmission.

The number of messages, delivered by each node per minute, for 10 minutes, was counted in order to analyze traffic by nodes, which was examined through simulation. Figure 3 shows the value of normal message transmission.

Sensor 1 and Sensor 2, which regularly generate nodes, created about 150 messages while Sensor 4 and 5 created a large number of messages in the initial stage of connection but, later, about 10 messages as the data become stabilized.

**Figure 4.**
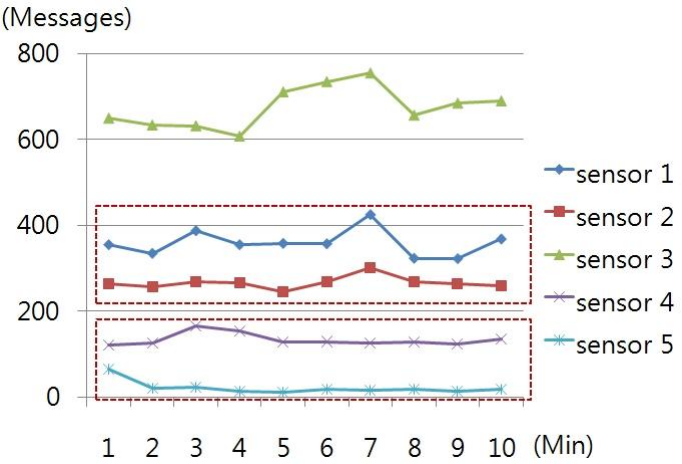
A sudden rise in the number of messages due to the attack from Sensor 3.

Figure 4 shows that the traffic of Sensor 2, 3, and 4 increases as Sensor 3 makes malicious data requests and transmission, creating about 60 messages, which is a 100% increase compared to the previous number of messages. However, Sensor 5, not affected by Sensor 3, sends a message with a regular pattern.

### 4.2. Behavior Data between Nodes

The node for the test-bed is TIP 700CM sensor, which senses illumination, humidity, and temperature with the following message format.

**Figure 5.**
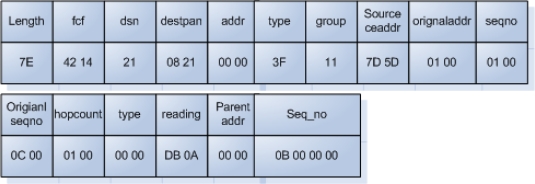
Sensor data format.

The normal data collected through the structure above has the following format depicted in Figure 5.

**Figure 6.**
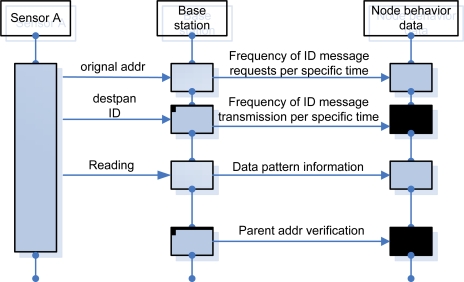
Process of verifying behaviors between nodes.

For the analysis of node behaviors, BS verifies the behaviors requested from each sensor and saves the data. Figure 6 shows the process of verifying and saving behaviors between nodes.

According to the result of the test-bed, Sensor 3 keeps requesting/sending data from/to Sensor 1, 2, and 4. By confirming messages of a sudden increase, we can detect which node requests a message and the ID of the sensor node generating unnecessary traffic.

**Figure 7.**
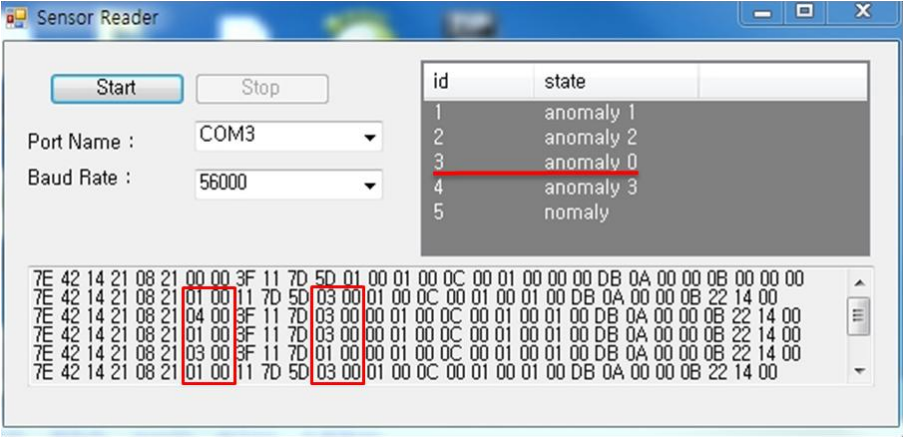
Detection of an abnormal node by node behaviors analysis.

Figure 7 shows the result of confirming an abnormal node by collecting data of the sensor nodes. Under ‘State’, ‘anomaly_number’ shows which node makes the greatest number of abnormal requests and we can see that the node of id 3, ‘anomaly 0’, makes the most frequent message requests and generation. The following numbers help to figure out whether or not each sensor makes malicious attacks or is being attacked. Therefore, based on this information, the system will report to the network manager and remove Sensor 3 from the service so as to monitor changes in other nodes.

## 5. Conclusions

In this research, we have suggested a DoS detection system based on global interdependent behaviors, which analyzes traffic and tracks node behaviors in a sensor network environment. Particularly, in the active sensor network, data traffic is analyzed with BS through message delivery patterns among nodes, and node behaviors are tracked by data format analysis. Based on the result, the entire data of behaviors are managed and rule information is generated, which helps to examine the conditions among nodes.

Through the test-bed we could detect a node making a malicious attack by discovering message behaviors among nodes and comparing message traffic from a sensor attempting a DoS attack. The research could be very useful for smart phones which can analyze irregular messages. It can be used to confirm unnecessary service connection to check unnecessary information.

The suggested method of detecting a DoS attack based on interdependent behaviors in the sensor network applies the steps required by the requirements of an effective detection system. By doing so, the system itself can block attacks to overcome a problem of errors and can report a precursor symptom to a manager to increase the stability of the system.

## Figures and Tables

**Table 1.** TIP 700CM sensor message format.

Header Description
Length (1 byte): message length in bytes not including header
Fcf (2 byte): IEEE 802.15.4 frame control field [reserved]
Dsn (1 byte): IEEE 802.15.4 data sequence number [reserved]
Destpan (2 byte): IEEE 802.15.4 Destination personal area network identifier [reserved]
Addr (2 byte): TinyOS destination address
Type(1 byte): TinyOS active message number
Group (1 byte): TinyOS group id
Multihop Message Description
Sourceaddr (2 bytes): address of the previous hop
Originaddr (2 bytes): address of the origin of the message
Seqno (2 bytes): sequence number of the previous hop of multihop messages
Originseqno (2 bytes): sequence number from the origin of multihop messages
Hopcount (2 bytes): hopcount
Surge Message Description
Type (2 bytes): type, type 0 is ‘sensor reading’
Reading (2 bytes): ADC reading from the sensor
Parentaddr (2 bytes): address of the parent in the multihop tree
Seq_no (4 bytes): sequence number of Surge messages
